# Supplementary material for: CLCa mediates a novel cross-talk between Wnt secretion and actin organization
Source: Life Sci Alliance. 2025 May 2;8(7):e202402962. doi: 10.26508/lsa.202402962 (PMC12050421; doi:10.26508/lsa.202402962)

Fig.1a

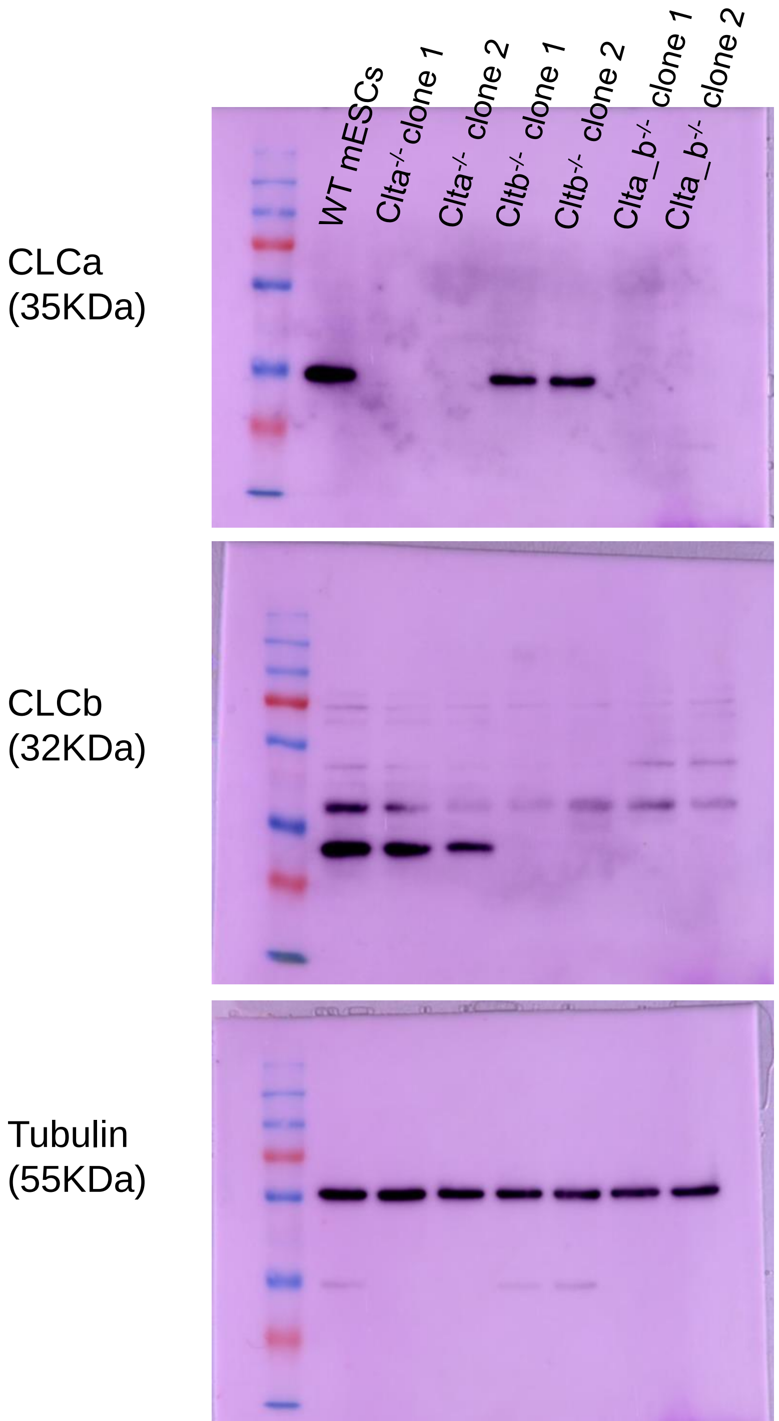

CLCa
(35KDa)
CLCb
(32KDa)
Tubulin
(55KDa)
WT mESCs
Clta-/- clone 1
Clta-/- clone 2
Cltb-/- clone 1
Cltb-/- clone 2
Clta_b-/- clone 1
Clta_b-/- clone 2

Fig3f

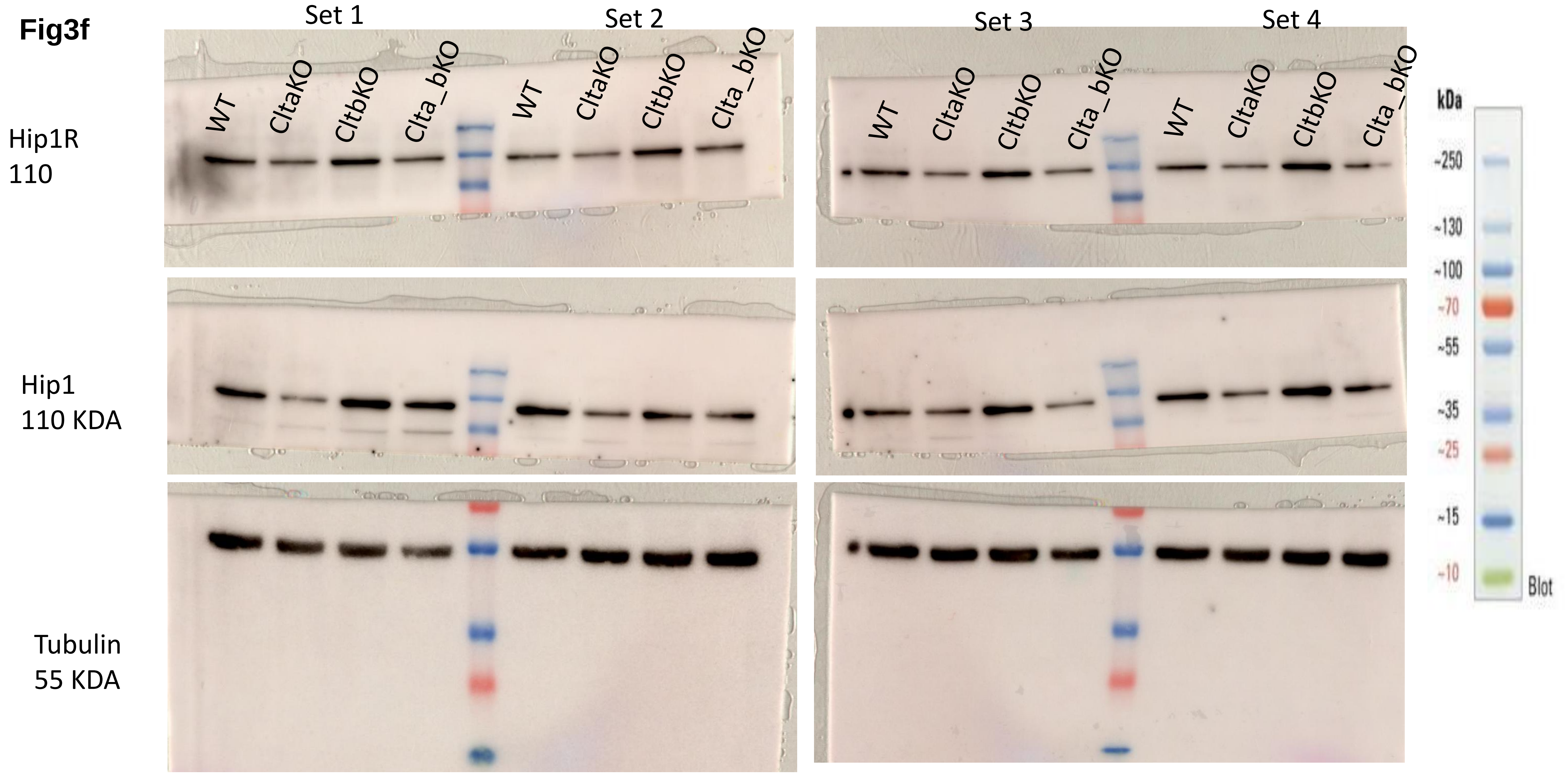

Hip1 probed after stripping Hip1R blot
Note- Hip1 and Hip1r raised in diff organisms
For WB Quantitation, Set 2, 3 and 4 were used

Fig3f

Set 1 Set 2 Set 3

Arp3
43kDa

Tubulin
55KDa

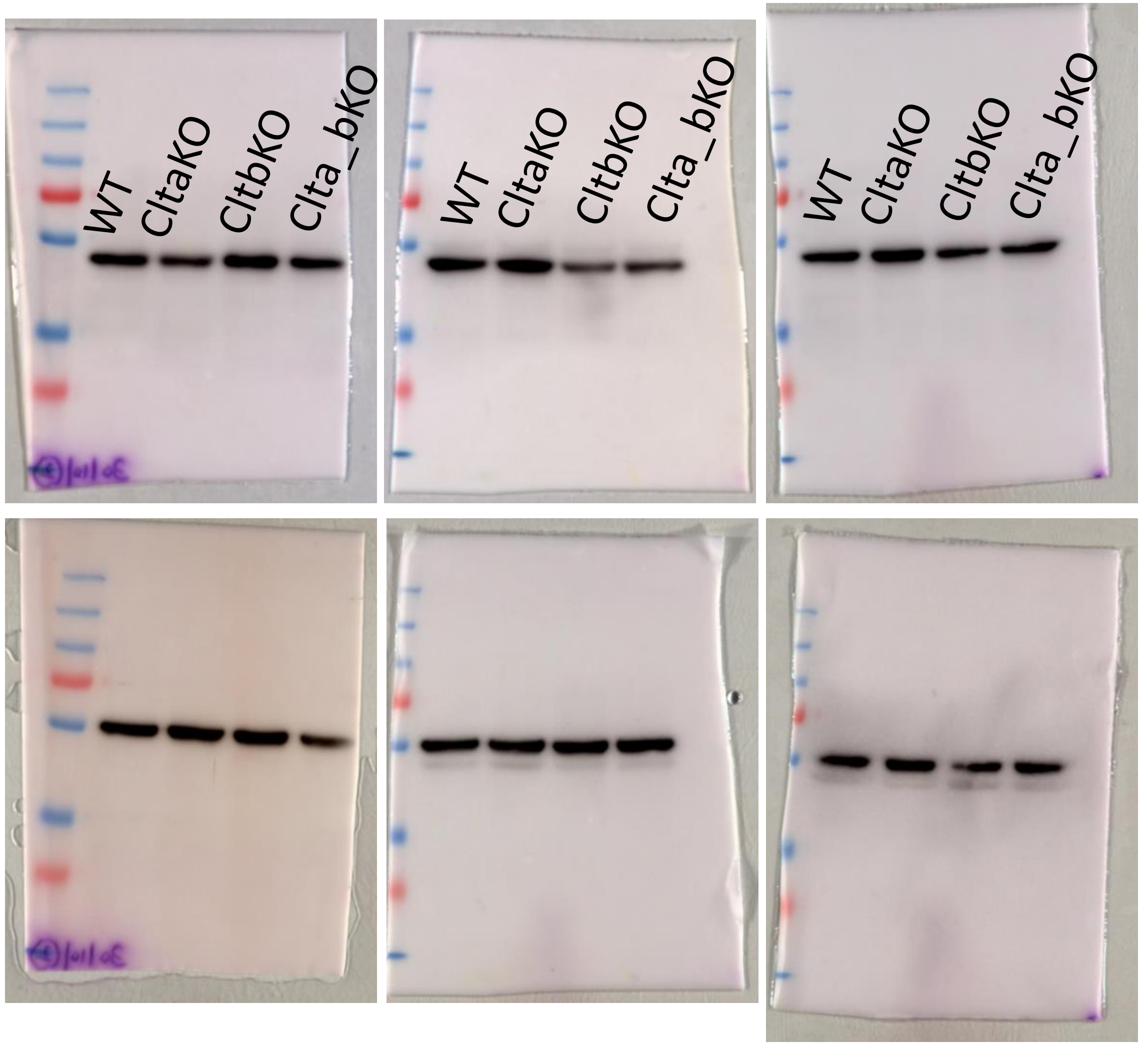

Fig3f

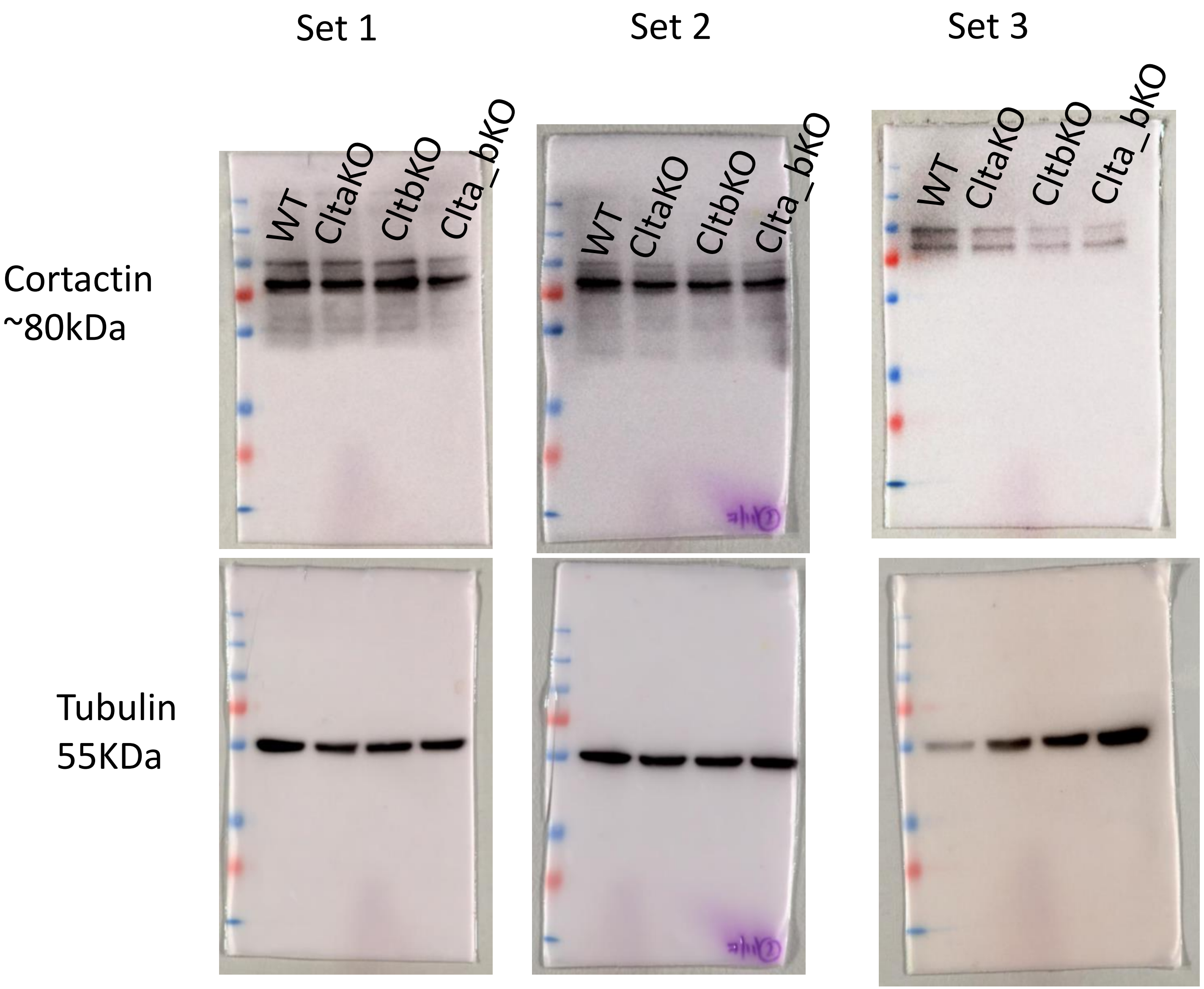

Set 1
Set 2
Set 3
WT
CltaKO
CltbKO
Clta_bKO
WT
CltaKO
CltbKO
Clta_bKO
WT
CltaKO
CltbKO
Clta_bKO
Cortactin
~80kDa
Tubulin
55KDa

Fig3f

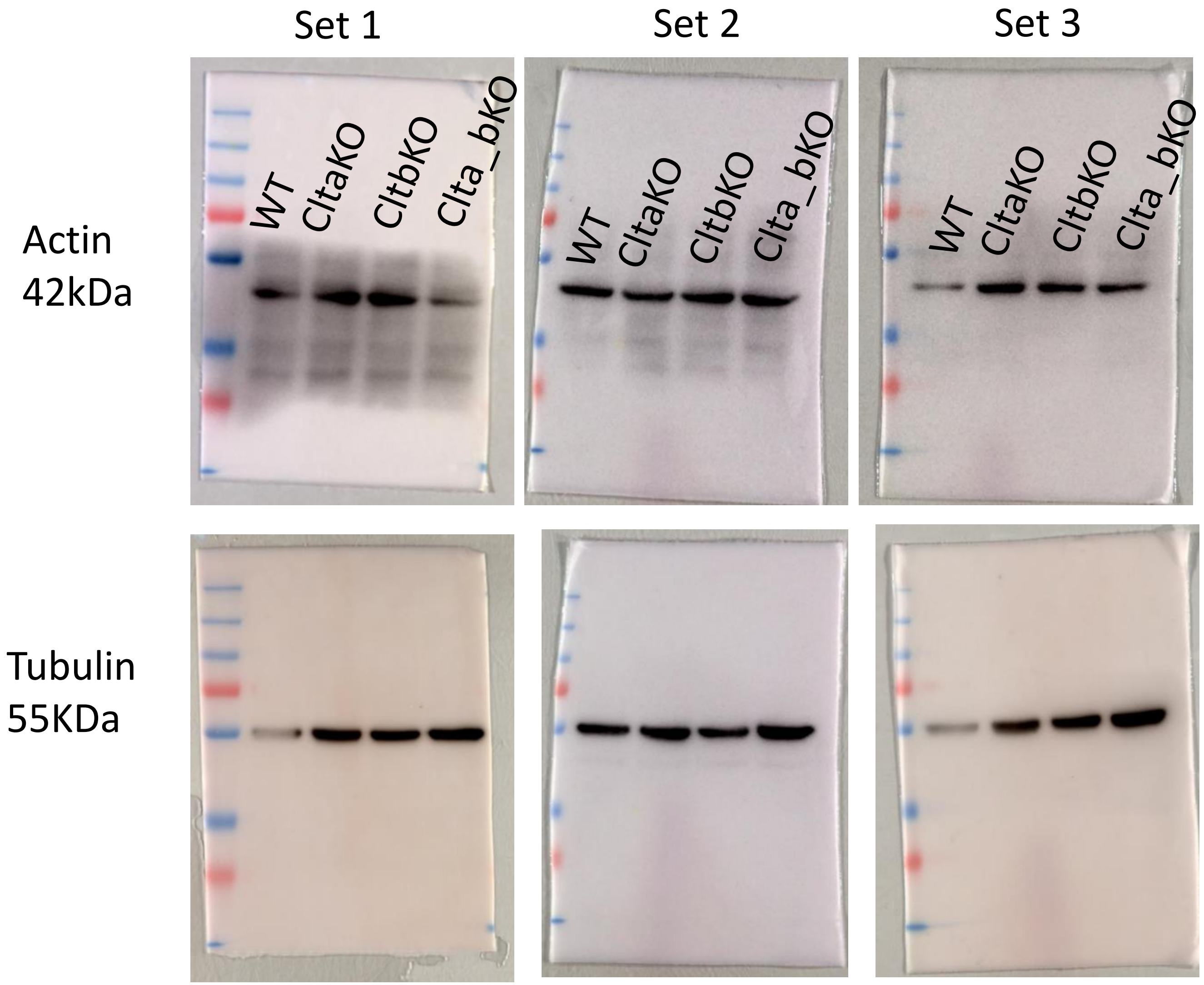
Set 1
Set 2
Set 3
Actin
42kDa
Tubulin
55KDa
WT
CltaKO
CltbKO
Clta_bKO

Supple Fig 6a

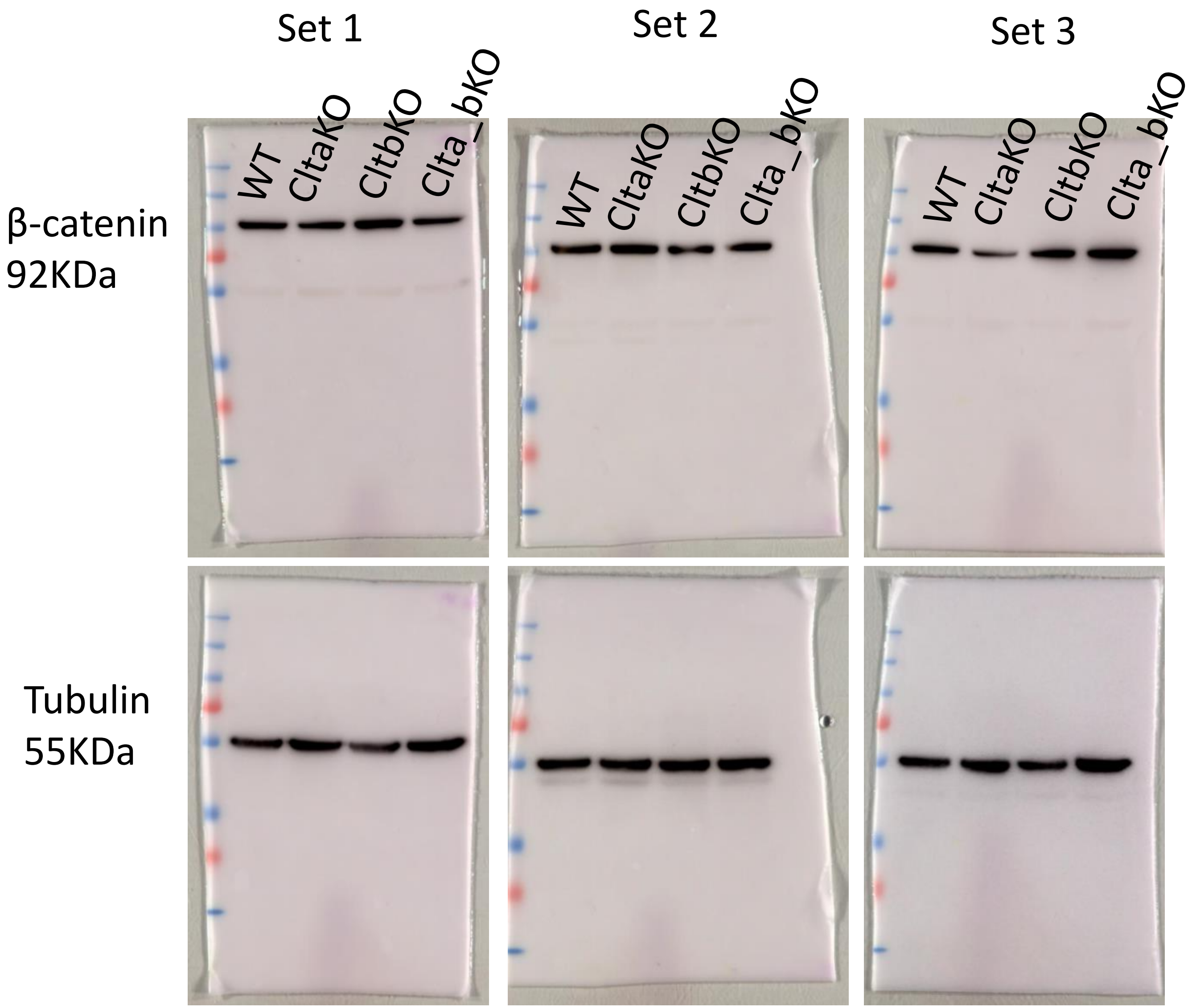

Supple Fig 7

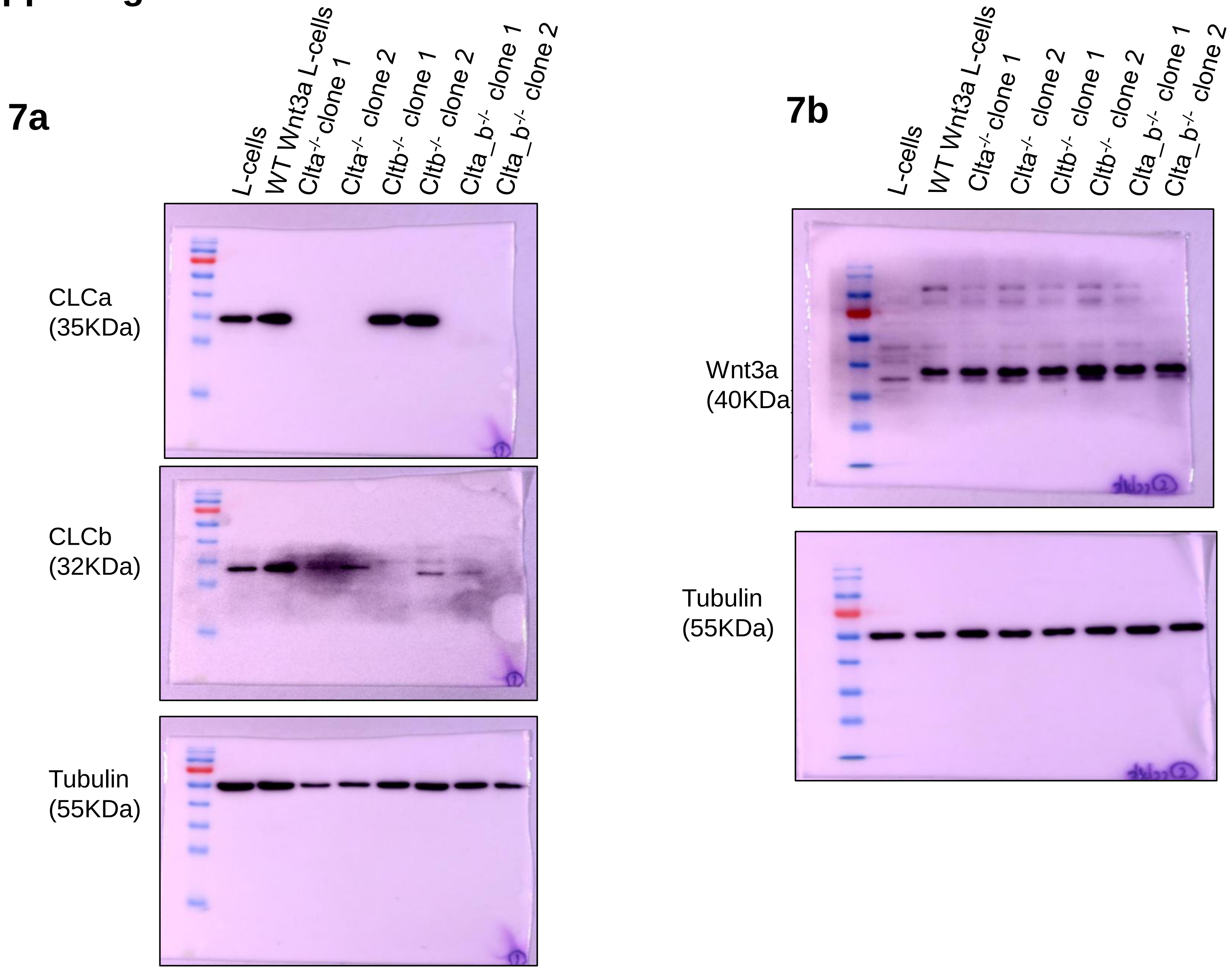
7a
L-cells
WT Wnt3a L-cells
Clta$^{-/-}$ clone 1
Clta$^{-/-}$ clone 2
Cltb$^{-/-}$ clone 1
Cltb$^{-/-}$ clone 2
Clta_b$^{-/-}$ clone 1
Clta_b$^{-/-}$ clone 2
CLCa (35KDa)
CLCb (32KDa)
Tubulin (55KDa)
7b
L-cells
WT Wnt3a L-cells
Clta$^{-/-}$ clone 1
Clta$^{-/-}$ clone 2
Cltb$^{-/-}$ clone 1
Cltb$^{-/-}$ clone 2
Clta_b$^{-/-}$ clone 1
Clta_b$^{-/-}$ clone 2
Wnt3a (40KDa)
Tubulin (55KDa)

Fig.5i

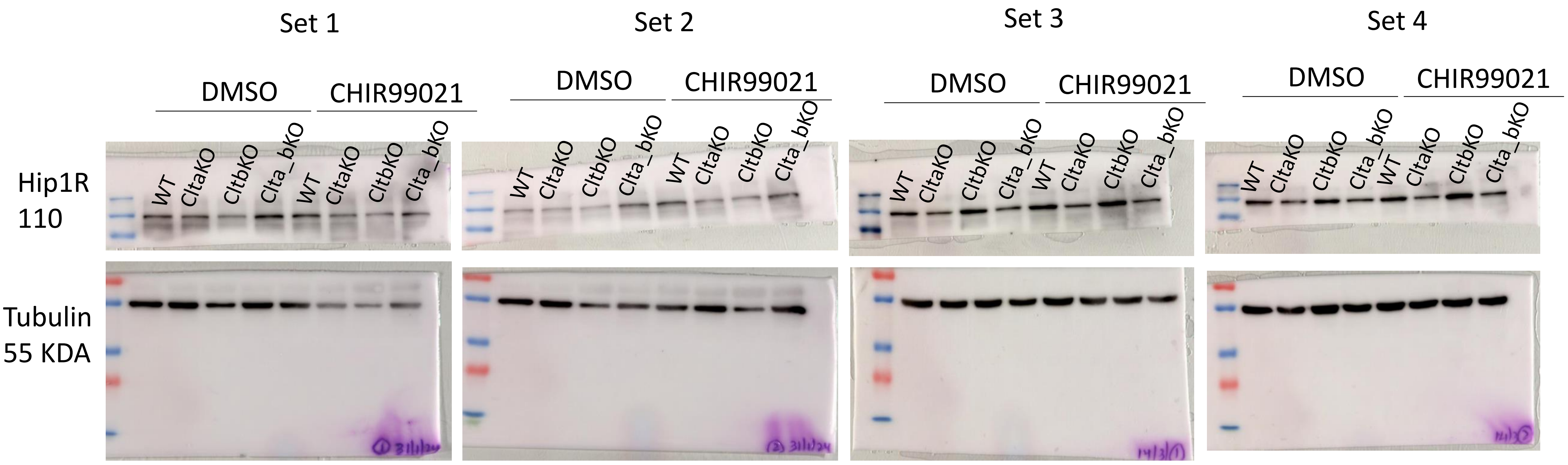
Set 1
Set 2
Set 3
Set 4
DMSO
CHIR99021
WT
CltaKO
CltbKO
Clta_bKO
Hip1R
110
Tubulin
55 KDA

Supple Fig 6c

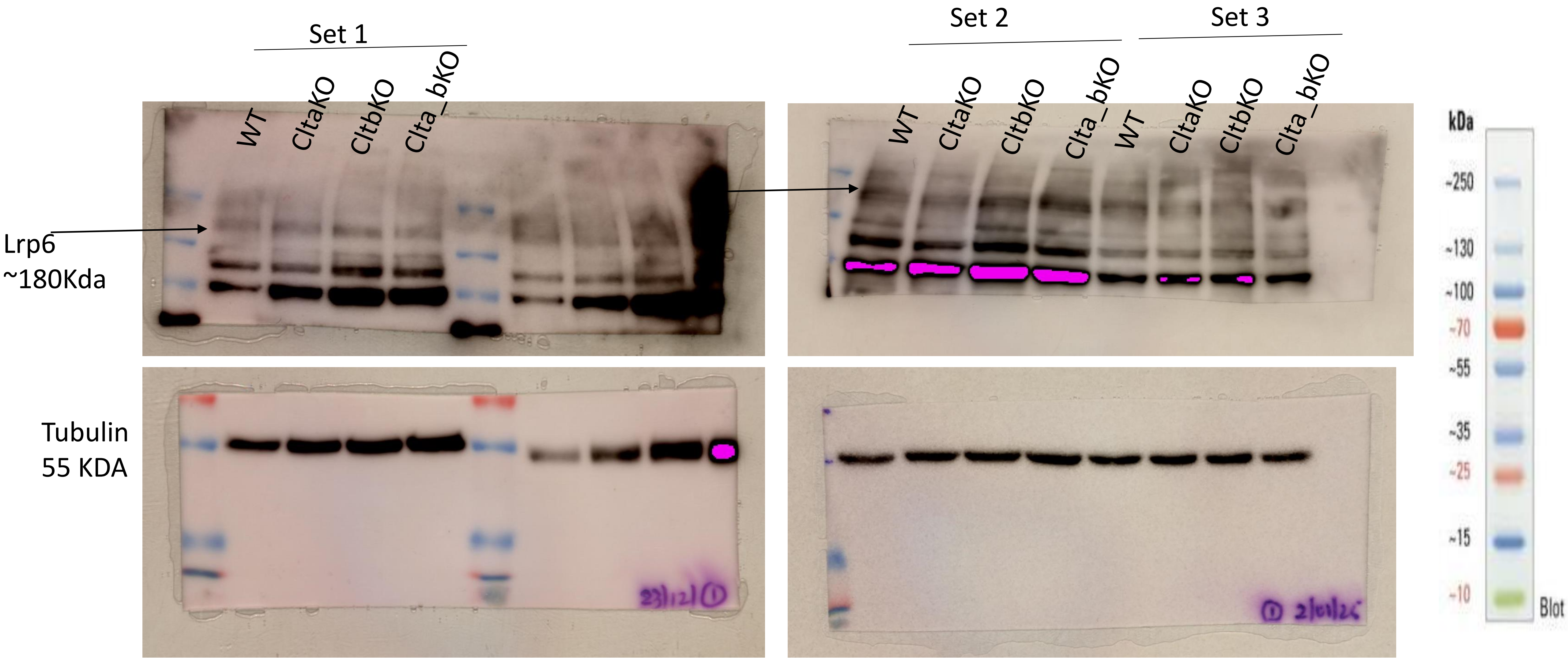

Supple Fig 10

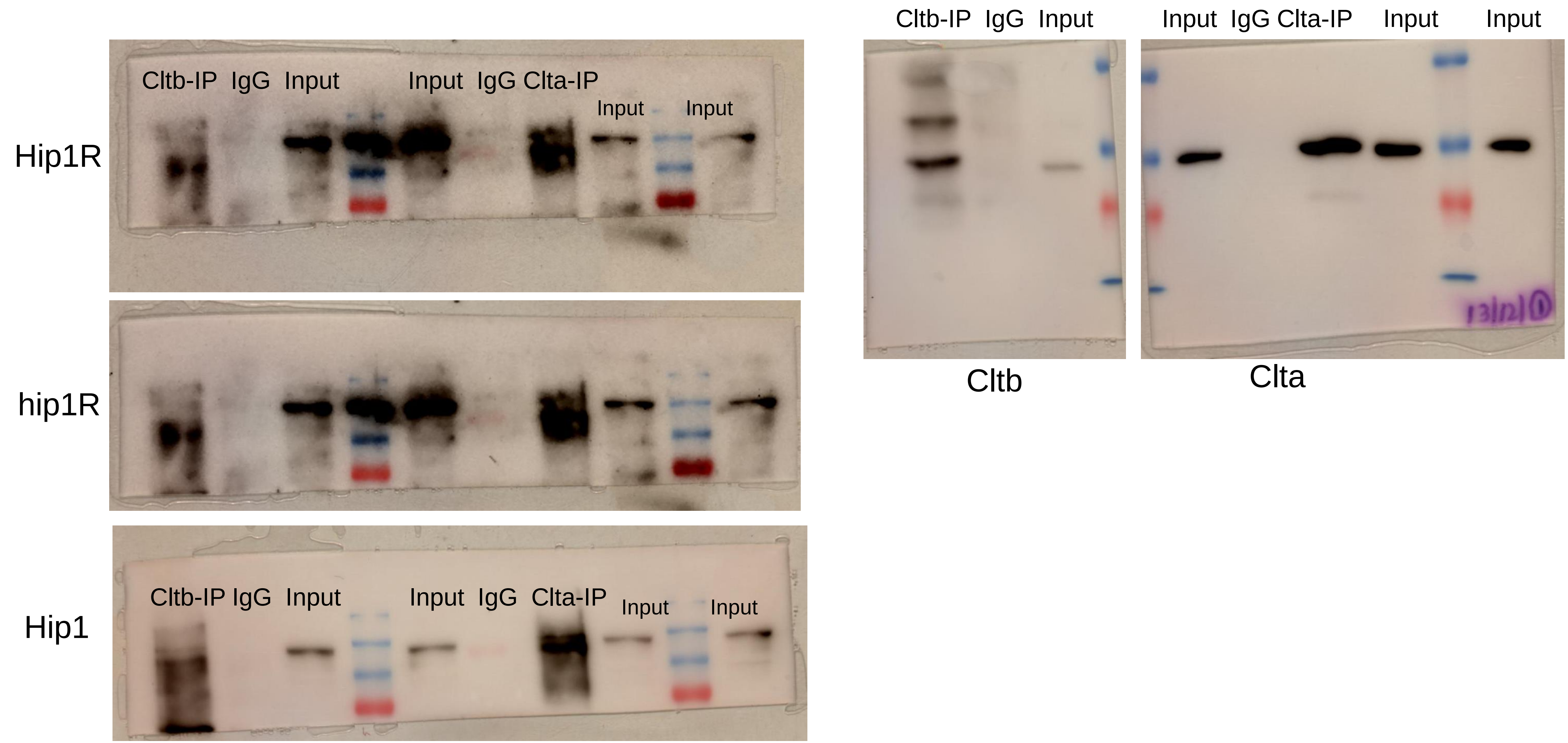

Supple Fig 11a

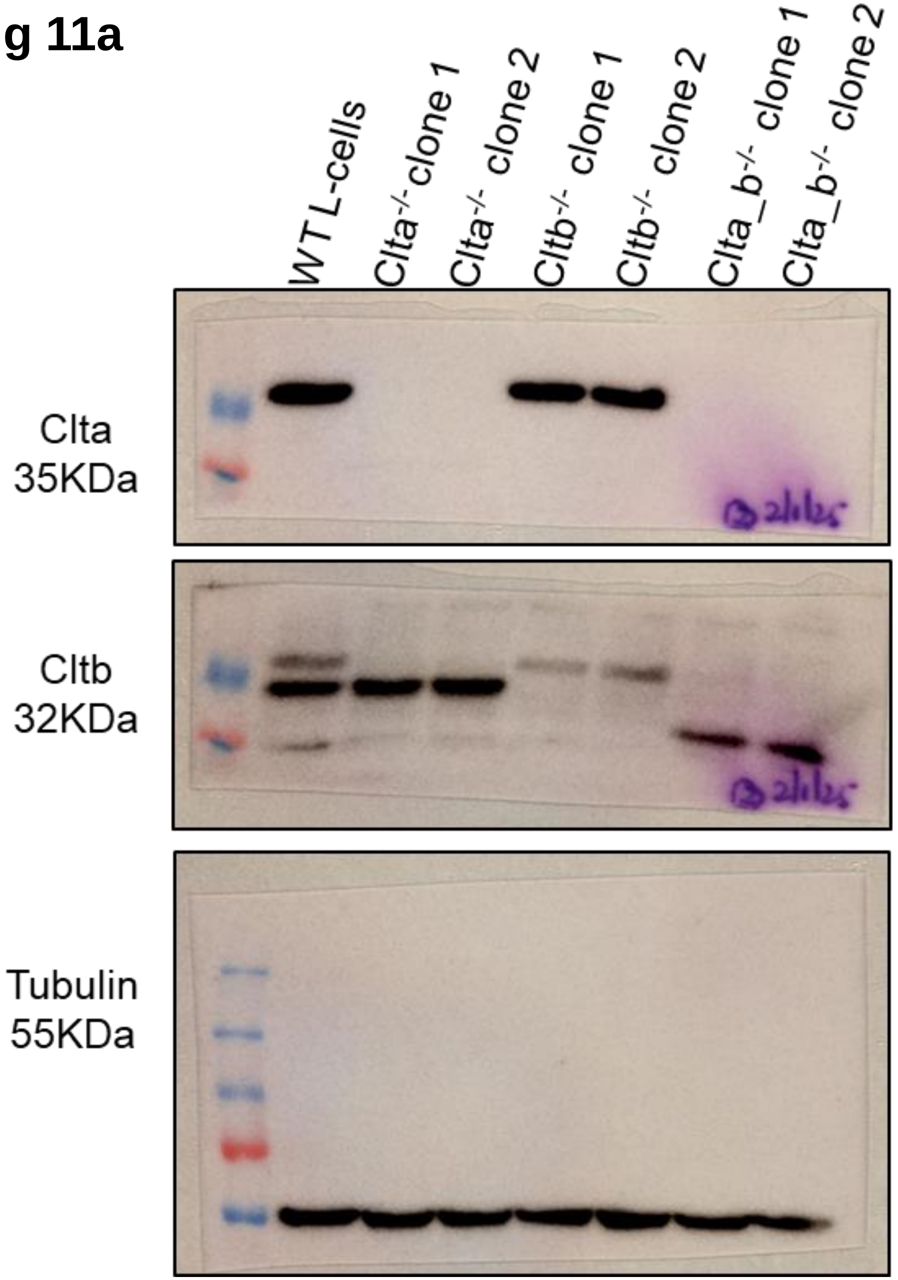

Supple Fig 14b

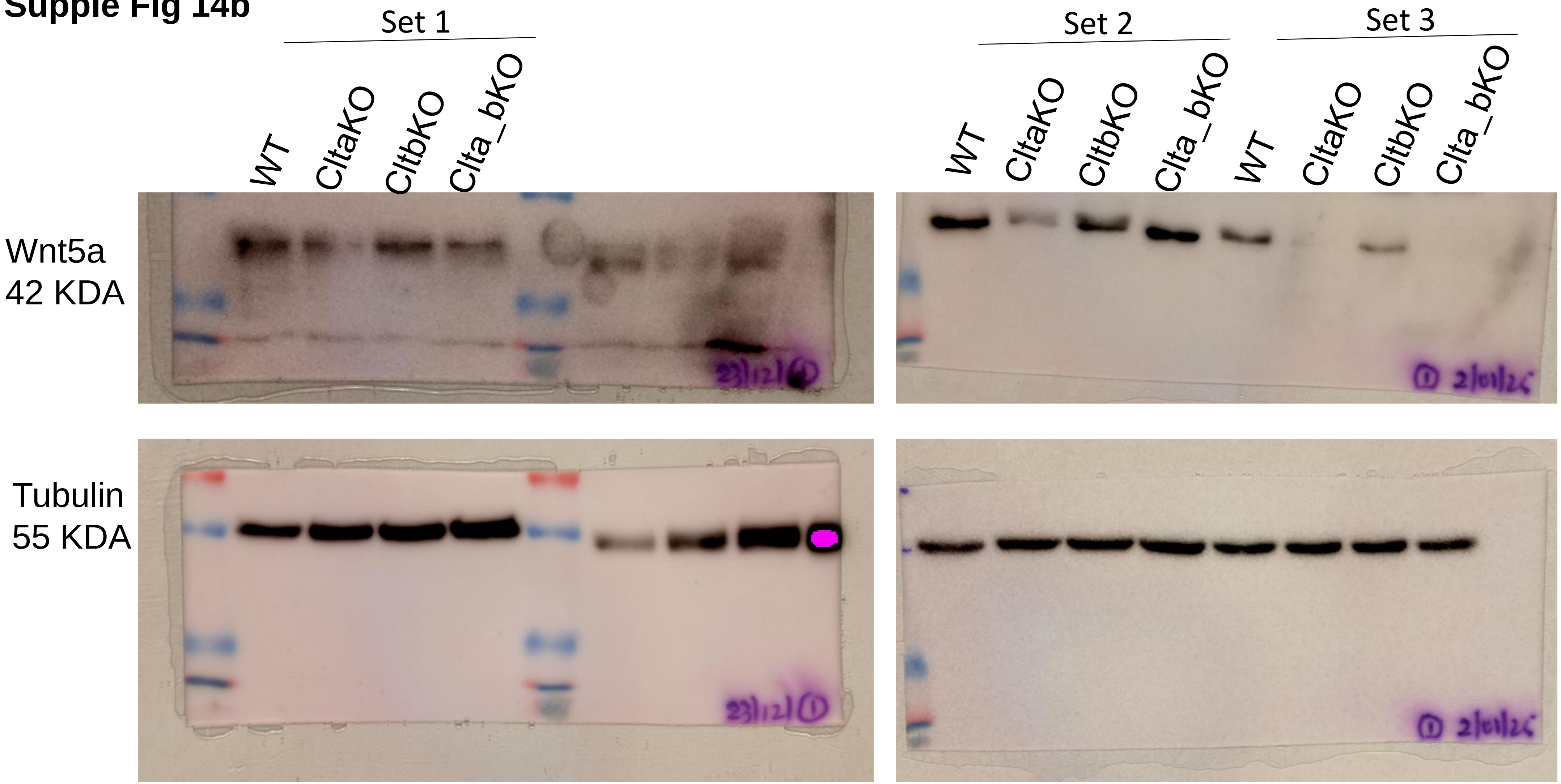

Supplement: Supplementary file 1 [file LSA-2024-02962_SdataF1.1_F3.1_F5.1_FS6.1_FS7_FS10_FS11_FS14.1.pdf]
